# Optimization of hydrothermal autoclaving parameters for the synthesis of porous ceramics from porcelain tile polishing residue

**DOI:** 10.1038/s41598-024-66125-7

**Published:** 2024-07-02

**Authors:** Liang Zhao, Shuang Yao, Jiayu Zhao

**Affiliations:** School of Civil and Architectural Engineering, Yangtze Normal University, Chongqing, 408100 China

**Keywords:** Porcelain tile polishing residue, Porous ceramic, Hydrothermal autoclaving, Pore structure, Materials science, Structural materials

## Abstract

Porous ceramics were synthesized using porcelain tile polishing residue (PTPR) and slaked lime (Ca(OH)_2_) as a reinforcing agent through a hydrothermal autoclaving method. The process parameters, including the quantity of slaked lime added, the hydrothermal autoclaving temperature, and the reaction duration, were optimized meticulously. The composition, structure, thermal and physical properties of the samples were thoroughly analyzed via Brunauer–Emmett–Teller (BET) measurements, powder X-ray diffraction (PXRD), and scanning electron microscopy (SEM). The results indicated that the incorporation of slaked lime and hydrothermal autoclaving led to the formation of calcium silicate hydrate, which corresponded with an enhancement in the strength of the sample. Notably, when the quantity of slaked lime added was optimized at 30 wt%, the formation of tobermorite (5CaO·6SiO_2_·5H_2_O) was detected. At a hydrothermal autoclaving temperature of 150 °C, the formation of only sheet-like calcium silicate hydrate was observed. In contrast, at an elevated temperature of 180 °C and 210 °C, needle-like tobermorite was successfully synthesized. The porous ceramic with the most favorable structure was obtained through autoclaving at 180 °C for 10 h with 30 wt% slaked lime, exhibiting a total pore volume of 0.11 mL/g, a specific surface area of 26.35 m^2^/g, and a mesoporous volume fraction of 90.40%.

## Introduction

Porcelain tiles, a widely sought-after building material, are extensively utilized in the industry for architectural decoration. Recent statistics indicate that, in 2023, China’s annual porcelain tile production reached an impressive 6.74 billion square meters^1^. During manufacturing, to achieve the desired flatness, thickness, and glossiness, sintered porcelain tiles undergo a polishing process to remove surface layers between 0.4 and 0.8 mm thick. This process generates a byproduct known as porcelain tile polishing residue (PTPR), which is categorized as industrial solid waste. It is estimated that the generation of PTPR amounts to approximately 0.6–0.8 kg per square meter of tiles produced^2^, culminating in an annual output exceeding 4 million tons in China. Currently, PTPR is predominantly managed through landfilling, which frequently leads to secondary pollution issues such as airborne dust contamination during transport and groundwater pollution from landfill seepage. This environmental pollution not only harms ecological systems but also hinders the sustainable development of the porcelain tile industry^3–5^. Consequently, the effective utilization of PTPR is crucial.

Researchers, including Ke^6^, Xian^7^, and Carmenlucia^8^, have investigated the recycling of polishing residue in porcelain tile production, enhancing its cost-effectiveness. The residue’s rich amorphous SiO_2_ and Al_2_O_3_ content has led Giovanny^9^, Luiz^10^, and Jacoby^11^ to use it as a cement substitute, contributing to composite Portland cement innovation. Lin^12^ has transformed ceramic polishing residue into a beneficial coating additive, enhancing polymer and white paint film qualities through optimized dispersion in water. Additionally, Wang^13^, Ji^14^, Niu^15^, and Kırsever^16^ have produced foam ceramics from porcelain tile polishing residue through high-temperature sintering, showing potential for novel building insulation materials. However, despite the escalating focus on the resourceful utilization of porcelain tile polishing residue in recent years, the fabrication of porous ceramics from such residue, particularly under non-sintering conditions, remains in a nascent stage of exploration. The hydrothermal autoclaving method is characterized by low energy consumption due to its mid-to-low temperature operations, broad material applicability, precise reaction condition control, and an environmentally benign enclosed system for managing toxic reactions^17^. These features indicate its substantial potential in non-sintering conditions.

In this research, porous ceramics were synthesized via hydrothermal autoclaving method, employing porcelain tile polishing residue as the primary raw material and slaked lime (Ca(OH)_2_) as a reinforcing agent. The composition, pore structure, and microstructure of the fabricated ceramics were thoroughly characterized through powder X-ray diffraction (PXRD), Brunauer–Emmett–Teller (BET) analysis, and scanning electron microscopy (SEM). The effect of hydrothermal autoclaving parameters (the amount of slaked lime added, the autoclaving temperature, and the reaction duration) on thermal and physical properties was analyzed through comprehensive balance method of orthogonal experiment design, which is adept at addressing multi-index optimization challenges, facilitating in harmonizing various objectives to derive an optimal, holistically performant solution^18–20^. This research not only establishes a theoretical foundation for the synthesis of porous ceramics as building insulation materials without sintering but also proposes an energy-efficient method for the utilization of porcelain tile polishing residue.

## Experimental method and process

### Raw materials

**Figure 1:**
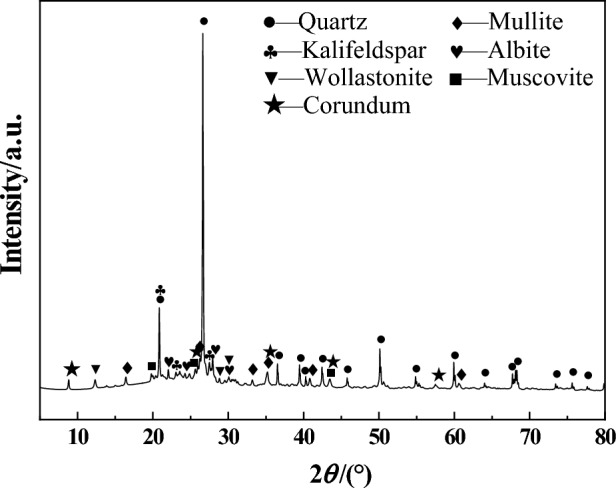
PXRD pattern of the polishing residue used in this study.

**Figure 2:**
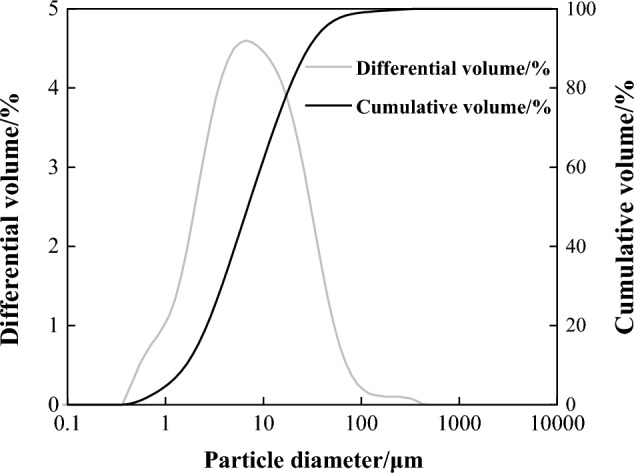
Particle size distribution of the PTPR.

The primary raw material employed in this research was porcelain tile polishing residue, sourced from a production line of 600 mm × 1200 mm polished tiles in Monalisa Group Co., Ltd, utilizing diamond as the abrasive. Table 1 elucidates its chemical composition. Figure 1 depicts the mineral composition, while Fig. 2 shows the particle size distribution, with D10, D50, and D90 values of 1.5 μm, 7.1 μm, and 29.3 μm, respectively. Analytical-grade slaked lime, procured from Tianjin Hongyan Chemical Company, served as the reinforcing agent.

**Table 1:** Chemical composition of the PTPR (wt.%) used in this study.

Composition	SiO_2_	Al_2_O_3_	Fe_2_O_3_	TiO_2_	CaO	MgO	K_2_O	Na_2_O	IL
PTPR	66.42	20.73	1.03	0.35	1.59	1.14	3.07	2.22	3.45

*IL* ignition loss.

### Sample fabrication

**Figure 3:**
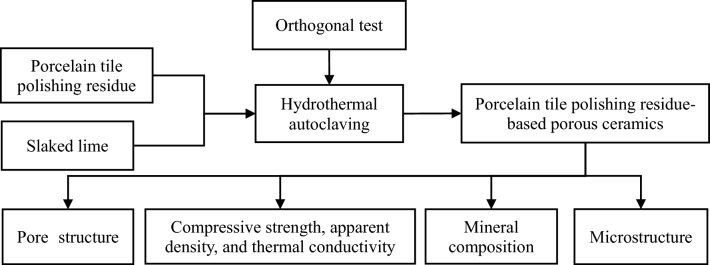
Overview of the methodology utilized to fabricate porous ceramic samples from PTPR.

Figure 3 illustrates the methodology utilized in producing porous ceramics from porcelain tile polishing residue. To fully react and form calcium silicate hydrate and tobermorite, the expected calcium to silicon ratio (Ca/Si) should be approximately 1.5 and 0.8, respectively^21,22^. However, such a high Ca/Si ratio is not essential, considering that porcelain tile polishing residue already possesses a porous structure. Thus, only a modest amount of CaO is required to produce a certain quantity of calcium silicate hydrate and tobermorite, which will enhance the mechanical properties of the specimen and improve its pore structure, ultimately maximizing the utilization of ceramic polishing waste. Consequently, the samples were synthesized by altering the slaked lime quantity—0, 10, 20, and 30 wt%—added to the porcelain tile polishing residue, designated as PTPR0, PTPR10, PTPR20, and PTPR30, respectively. After homogenous stirring of the reaction mixture, 10wt% water was incorporated for granulation. This was followed by an aging process lasting 12 h to ensure moisture uniformity, then molding at 25 MPa pressure, and subsequent drying. The resultant green bodies were then placed in a reactor and subjected to hydrothermal autoclaving at diverse temperatures (150, 180, or 210 °C) for varying durations (7, 10, or 13 h).

A three-factor, three-level orthogonal test design, L_9_(3^3^), was employed to analyze the influence of three variables—the quantity of slaked lime added, the hydrothermal autoclaving temperature, and the reaction duration—on the compressive strength and apparent porosity of the samples. Table 2 outlines the orthogonal test design parameters.

**Table 2:** Factor levels of the orthogonal tests.

Level	Factor A	Factor B	Factor C
	Quantity of slaked lime added/%	Hydrothermal autoclaving temperature/°C	Reaction duration/h
1	10	150	7
2	20	180	10
3	30	210	13

### Performance characterization

The apparent porosity of the samples was determined using Archimedes’ principle in water, as outlined in Eq. (1):

1 $$ P = \, \left( {m_{{3}} - m_{{1}} } \right)/\left( {m_{{3}} - m_{{2}} } \right) \, \times { 1}00\% , $$

where *m*_1_ is the mass of the dry sample, *m*_2_ is the apparent mass of the saturated sample in water, and *m*_3_ is the mass of the saturated sample in air.

The mechanical load that the samples can withstand was measured using a testing machine (CSS44100), at a loading rate of 0. 5 kN/s. The compressive strength was calculated using Eq. (2):

2 $$ P = F/S, $$

where *F* is the maximum load and *S* is the area of the force applied to the sample.

The thermal conductivity of the samples was measured using a thermal conductivity tester (Xiangtan Xiangyi Instrument Co., Ltd., DRH-III) was used. In the test, the temperatures of the hot and cold surfaces were maintained at 35 °C and 25 °C, respectively, with the sample dimensions being 300 mm × 300 mm × 30 mm.

The mineral composition of the sample was analyzed using an X-ray diffractometer (MDI, WJP75-91WJQ9). The micromorphology of the samples was examined using a scanning electron microscope (FEI, Quanta 600FEG). The pore size distribution and characteristic parameters of the samples were determined using an automatic surface and porosity analyzer (McMurray Instrument Co., Ltd, ASAP2460).

## Results and discussion

### Compressive strength, apparent density, and thermal conductivity of porcelain tile polishing residue-based porous ceramics

Systematic orthogonal tests facilitated the preparation of various samples under specific hydrothermal autoclaving conditions. The indicators (compressive strength, apparent density, and thermal conductivity) of the samples were subsequently quantified. The results are presented in Table 3, where ‘A’ indicates the amount of slaked lime added, ‘B’ the autoclaving temperature, and ‘C’ the reaction duration.

**Table 3:** Results of orthogonal tests on the samples prepared in this work.

No	A	B	C	Compressive strength/MPa	Apparent porosity/%	thermal conductivity/W·(m·K)^-1^
1	10	150	7	6.3	45	0.065
2	10	180	10	7.9	46	0.052
3	10	210	13	13.5	44	0.069
4	20	150	10	15.8	44	0.064
5	20	180	13	17.9	44	0.062
6	20	210	7	20.1	44	0.060
7	30	150	13	19.4	44	0.058
8	30	180	7	18.8	43	0.063
9	30	210	10	41.6	46	0.049

Table 3 illustrates that the compressive strength of the specimens exhibits considerable variation across different factors and levels, achieving a maximum of 41.6 MPa. This strength is adequate for application in certain load-bearing wall structures. Conversely, the thermal conductivity demonstrates minimal fluctuation, ranging from 0.049 to 0.069, which is significantly lower than the maximum thermal conductivity of ≤ 0.23 required for building insulation materials. Furthermore, these values comply with the Chongqing region’s building energy conservation standard of 65%.

**Figure 4:**
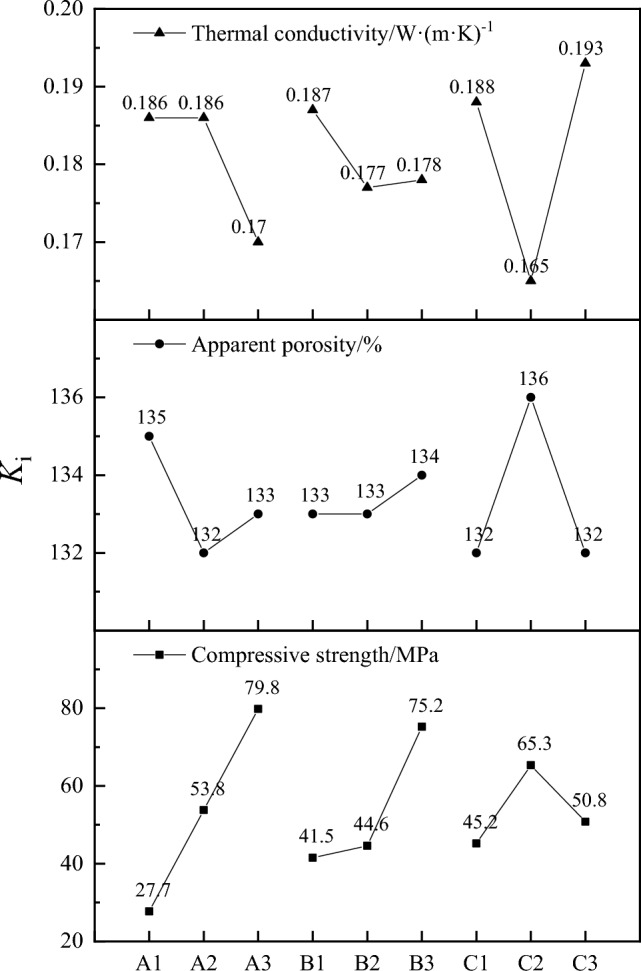
Relationship between each factor, level, and K value.

Table 4 is the result of calculations performed on Table 3 using a comprehensive balance method. The findings are exhibited in Table 4, where *K*_i_ represents the sum of indicators measured at an identical level for each factor, and ∆*R* denotes the range of the sum of *K*_i_. Figure 4 facilitated the elucidation of the relationship among each factor, level, and *K* value, as depicted in.

**Table 4:** Analysis of the experimental results for the samples prepared in this study.

	Compressive strength/MPa			Apparent porosity/%			Thermal conductivity/W·(m·K)^−1^		
	A	B	C	A	B	C	A	B	C
*K*_1_	27.7	41.5	45.2	135	133	132	0.186	0.187	0.188
*K*_2_	53.8	44.6	65.3	132	133	136	0.186	0.177	0.165
*K*_3_	79.8	75.2	50.8	133	134	132	0.170	0.178	0.193
*∆R*	52.1	33.7	20.1	3	1	4	0.016	0.010	0.028

For instance, in analyzing compressive strength values, the compressive strength value corresponding to (*K*_A1_) is 27.7 MPa, which is the sum of the compressive strength results measured for factor A (the quantity of slaked lime added) at the same level 1 (10wt%): 6.3 MPa + 7.9 MPa + 13.5 MPa = 27.7 MPa. The range (*∆R*_A_) is the difference between the maximum and minimum values among (*K*_A1_) (27.7 MPa), (*K*_A2_) (53.8 MPa), and (*K*_A3_) (79.8 MPa): 79.8—27.7 = 52.1 MPa. A larger *∆R* indicates a greater impact of this factor on compressive strength. Because of *∆R*_A_(52.1 MPa) > *∆R*_B_(33.7 MPa) > *∆R*_C_(20.1 MPa), the order of influence on compressive strength is: quantity of slaked lime added (A) > hydrothermal autoclaving temperature (B) > reaction duration (C).

For apparent porosity and thermal conductivity, the order is: reaction duration (C) > amount of slaked lime added (A) > hydrothermal autoclaving temperature (B). Distinct optimal solutions emerged for the three properties; for compressive strength, the optimal solution is A3B3C2, and for apparent porosity and thermal conductivity, it is A1B3C2. Considering that A3 yields the highest strength, moderate porosity, and thermal conductivity, and A1 the lowest strength, the comprehensive analysis of optimal parameters for compressive strength, apparent porosity, and thermal conductivity led to the selection of A3B3C2 as the optimal solution. This corresponds to adding 30 wt% slaked lime, a hydrothermal autoclaving temperature of 210 °C, and a reaction duration of 10 h.

### Pore structure characteristics of porcelain tile polishing residue-based porous ceramics

**Figure 5:**
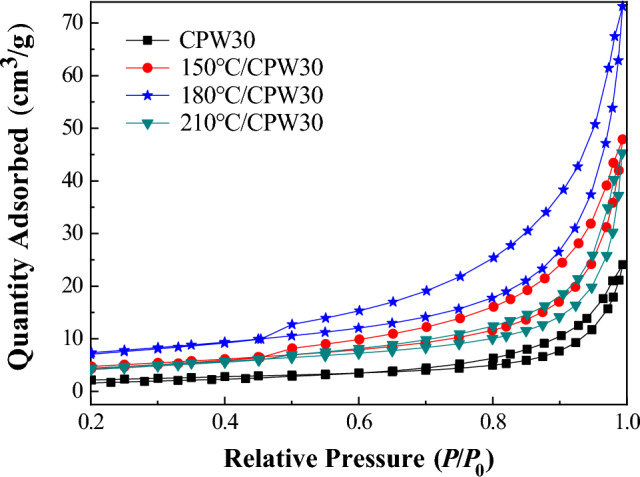
Nitrogen adsorption–desorption isotherms of PTPR30 samples prepared at different hydrothermal autoclaving temperatures.

The hydrothermal autoclaving temperature exhibited a minimal influence on the compressive strength, apparent porosity, and thermal conductivity. In order to further determine the optimal experimental parameters of hydrothermal autoclaving temperatures, nitrogen adsorption measurements were carried out to characterize the pore structures of the samples prepared at different hydrothermal autoclaving temperatures for 10 h with the addition of 30 wt% slaked lime. Nitrogen adsorption–desorption isotherms of the PTPR30 samples, synthesized under these conditions, are depicted in Fig. 5.

Figure 5 demonstrates that the isotherms exhibit typical type II adsorption characteristics^23^. The nitrogen adsorption–desorption isotherms of the PTPR, with slaked lime added before and after hydrothermal autoclaving, display distinct profiles, indicating the presence of desorption hysteresis. A pronounced ‘hysteresis loop’ emerges when the relative pressure *P/P*_0_ surpasses 0.45, with the hysteresis area of the PTPR30 sample being most extensive at a hydrothermal autoclaving temperature of 180 °C. Consequently, it is evident that, following hydrothermal autoclaving, the PTPR30 sample exhibits mesoporous characteristics.

Regarding the polishing residue amended with slaked lime, the nitrogen adsorption–desorption isotherms of the samples show divergence pre- and post-hydrothermal autoclaving, indicative of desorption hysteresis. A marked hysteresis loop becomes evident when the relative pressure *P/P*_0_ surpasses 0.45, with the hysteresis area being most significant for the PTPR30 sample at a hydrothermal autoclaving temperature of 180 °C. Consequently, it is apparent that the PTPR30 sample acquires mesoporous properties following autoclaving.

Table 5 delineates the characteristic parameters of the pore structures of the PTPR30 samples, ascertained at different hydrothermal autoclaving temperatures via the BET method.

**Table 5:** Characteristic parameters of the pore structures of the PTPR30 samples at different hydrothermal autoclaving temperatures measured using the BET method.

Sample	Total pore volume (mL/g)	Specific surface area (m^2^/g)	Average pore diameter (nm)	Mesoporous volume percentage (%)		
				< 2 nm	2–50 nm	> 50 nm
PTPR30	0.03	7.89	15.09	0.33	82.09	17.58
150℃/PTPR30	0.07	17.23	12.63	0.01	89.24	10.75
180℃/PTPR30	0.11	26.35	12.67	0	90.40	9.60
210℃/PTPR30	0.07	16.09	16.67	0	75.41	24.59

As demonstrated in Table 5, an increase in the hydrothermal autoclaving temperature initially leads to an augmentation of the total pore volume and specific surface area, followed by a subsequent decline. Notably, at 180 °C, the PTPR30 sample exhibits the highest specific surface area, measured at 26.35 m^2^/g, coupled with a pore volume of 0.11 mL/g, an average pore diameter of 12.67 nm, and a mesoporous volume percentage of 90.40%. Remarkably, the specific surface area of this sample is 3.3 times that of the sample that underwent no hydrothermal autoclaving, and its total pore volume has also increased 2.7 times, signifying that hydrothermal autoclaving induces a significant pore expansion effect. Table 5 demonstrates that the pore volume reaches its zenith at 180 °C, resulting in the maximal apparent porosity observed macroscopically. In contrast, elevating the temperature to 210 °C diminishes the pore volume, indicating that the apex of apparent porosity is not achieved at the highest autoclaving temperature, but rather within a specific thermal range, proximate to 180 °C.

Integrating the data from Table 5 with the outcomes of the orthogonal tests, the optimal experimental parameters to achieve a material with superior physical properties—specifically, maximal porosity and mesoporous volume—were identified. The ideal conditions include a hydrothermal autoclaving temperature of 180 °C, a reaction duration of 10 h, and the addition of 30 wt% slaked lime.

### Mineral composition of porcelain tile polishing residue-based porous ceramics

**Figure 6:**
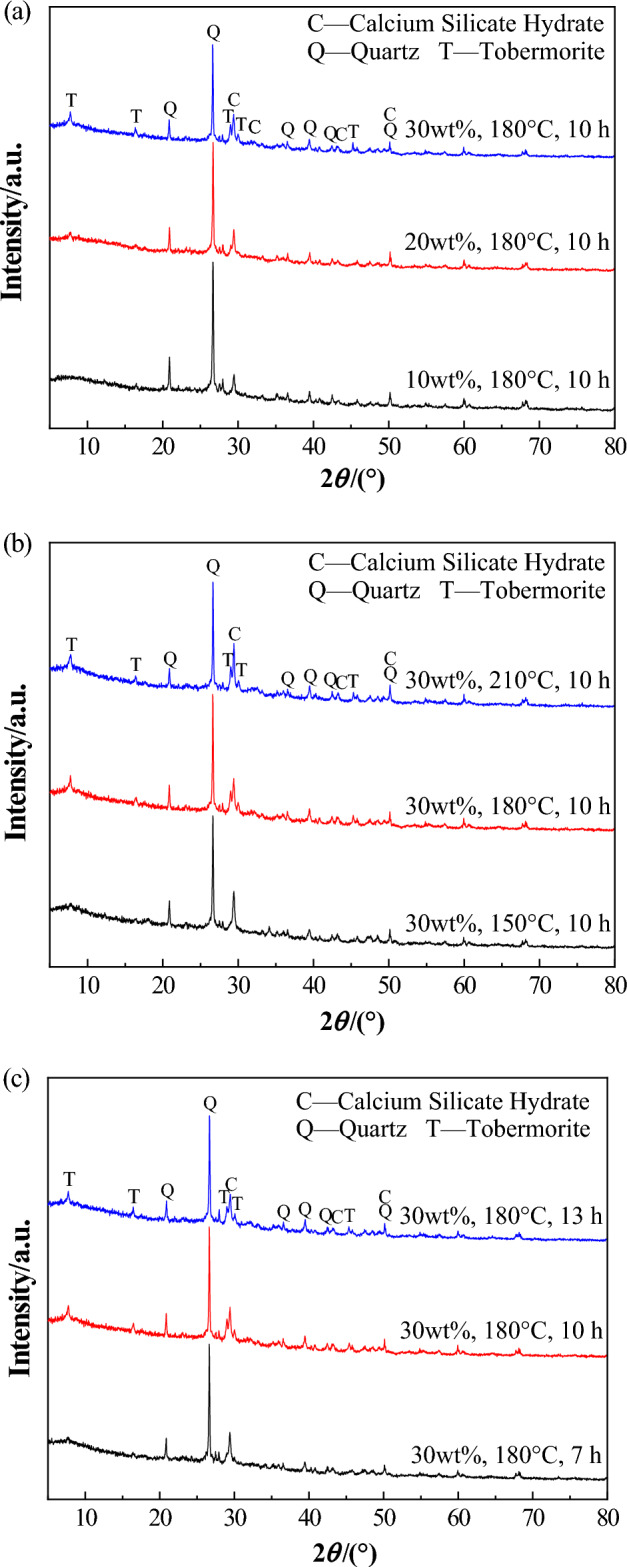
PXRD patterns of the samples prepared under different conditions. (**a**) different quantities of slaked lime added, at 10, 20, and 30 wt%, (**b**) different hydrothermal autoclaving temperatures, at 150, 180, and 210 °C, and (**c**) different reaction durations of 7, 10, and 13 h on the samples.

The phase compositions of the samples prepared under various reaction conditions were analyzed using powder X-ray diffraction. The results of these analyses are presented in Fig. 6.

Figure 6a displays the PXRD patterns of hydrothermally autoclaved samples with varying slaked lime content. Post-hydrothermal autoclaving, the distinct peak of calcium silicate hydrate is prominently observed at 2*θ* = 29.4°, indicative of the reaction between SiO_2_ from the porcelain tile polishing residue and Ca(OH)_2_ under hydrothermal conditions^24^. The presence of tobermorite (5CaO·6SiO_2_·5H_2_O) is evident with the addition of 20 wt% slaked lime. Furthermore, an increase in slaked lime content correlates with a heightened intensity of the tobermorite peak in the samples. This observation, coupled with the compressive strength data, implies that slaked lime promotes phase development and strengthens the samples^25^. As depicted in Fig. 6b, at 150 °C, only calcium silicate hydrate is formed, whereas the tobermorite peak appears when the hydrothermal autoclaving temperature exceeds 180 °C. Figure 6c reveals that over a period of 7–13 h, the characteristic peak intensity of tobermorite reaches its maximum value at 10 h, indicating that it is the optimal reaction duration conducive to the formation of this phase.

### Microstructure of porcelain tile polishing residue-based porous ceramics

**Figure 7:**
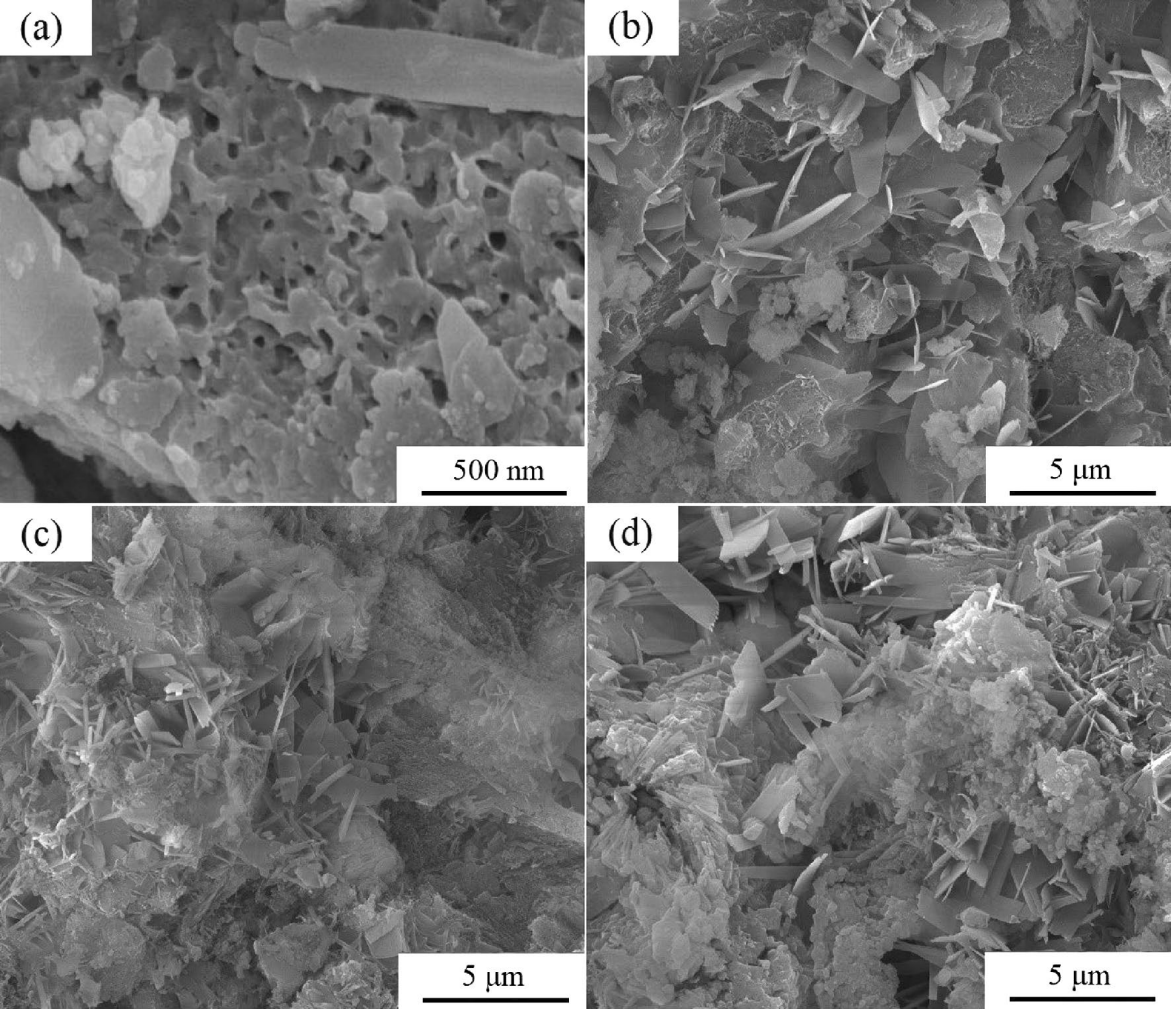
SEM morphologies of the PTPR30 samples prepared at different hydrothermal autoclaving temperatures. (**a**) Without hydrothermal autoclaving, (**b**) after hydrothermal autoclaving at 150 °C, (**c**) after hydrothermal autoclaving at 180 °C, and (**d**) after hydrothermal autoclaving at 210 °C.

The PTPR30 samples were observed by SEM at different hydrothermal autoclaving temperatures, the microstructures of which are shown in Fig. 7.

Figure 7a depicts the sample surface exhibiting a porous morphology. The BET results corroborate that materials from porcelain tile polishing residue inherently exhibit a porous structure, even before autoclaving. Figure 7b reveals that at a hydrothermal autoclaving temperature of 150 ℃, the reaction between SiO_2_ from the porcelain tile polishing residue and slaked lime leads to the formation of calcium silicate hydrate, characterized by sheet-like particles, which markedly improves the samples’ strength. Figure 7c demonstrates that at a hydrothermal autoclaving temperature of 180 °C, tobermorite forms with a needle-like structure. This phase not only enhances the strength but also significantly increases the porosity of the samples, as confirmed by the pore volume data in Table 5. In contrast, Fig. 7d shows that at a temperature of 210 °C, the ongoing production of calcium silicate hydrate results in a denser structure with improved strength, yet with reduced porosity, as indicated by the lower pore volume in Table 5.

## Conclusions

Upon reaching a slaked lime content of 30 wt% in the reactions, and setting the hydrothermal autoclaving temperature and duration to 180 °C and 10 h respectively, the resulting porcelain tile polishing residue-based porous ceramic exhibits optimal physical properties and pore structure. This is substantiated by a total pore volume of 0.11 mL/g, a specific surface area of 26.35 m^2^/g, and a mesoporous volume fraction of 90.40%.

After the addition of slaked lime and hydrothermal autoclaving, a novel calcium silicate hydrate phase materializes within the porous ceramic. When the amount of lime added increases from 20wt% to 30wt%, characteristic peaks of tobermorite appear in the sample, correlating with an enhancement in sample strength. At an autoclaving temperature of 150 °C, only calcium silicate hydrate with a sheet-like structure form. In contrast, temperatures of 180 °C or above induce the formation of a tobermorite phase with a needle-like structure. The peak intensity of the tobermorite is observed to culminate at a reaction duration of 10 h.

## Data Availability

The datasets used and/or analysed during the current study available from the corresponding author on reasonable request.
